# Voxel-wise analysis of dynamic ^18^F-FET PET: a novel approach for non-invasive glioma characterisation

**DOI:** 10.1186/s13550-018-0444-y

**Published:** 2018-09-10

**Authors:** Lena Vomacka, Marcus Unterrainer, Adrien Holzgreve, Erik Mille, Astrid Gosewisch, Julia Brosch, Sibylle Ziegler, Bogdana Suchorska, Friedrich-Wilhelm Kreth, Jörg-Christian Tonn, Peter Bartenstein, Nathalie Lisa Albert, Guido Böning

**Affiliations:** 1 Department of Nuclear Medicine, University Hospital, LMU Munich, Marchioninistr. 15, 81377 Munich, Germany; 2 German Cancer Consortium (DKTK), Partner site Munich, German Cancer Research Center (DKFZ), Heidelberg, Germany; 3 Department of Neurosurgery, University Hospital, LMU Munich, Munich, Germany

**Keywords:** FET PET, Glioma, Histogram analysis, *IDH* mutation

## Abstract

**Background:**

Glioma grading with dynamic ^18^F-FET PET (0–40 min p.i.) is typically performed by analysing the mean time-activity curve of the entire tumour or a suspicious area within a heterogeneous tumour. This work aimed to ensure a reader-independent glioma characterisation and identification of aggressive sub-volumes by performing a voxel-based analysis with diagnostically relevant kinetic and static ^18^F-FET PET parameters.

One hundred sixty-two patients with a newly diagnosed glioma classified according to histologic and molecular genetic properties were evaluated. The biological tumour volume (BTV) was segmented in static 20–40 min p.i. ^18^F-FET PET images using the established threshold of 1.6 × background activity. For each enclosed voxel, the time-to-peak (TTP), the late slope (Slope_15–40_), and the tumour-to-background ratios (TBR_5–15,_ TBR_20–40_) obtained from 5 to 15 min p.i. and 20 to 40 min p.i. images were determined. The percentage portion of these values within the BTV was evaluated with percentage volume fractions (PVFs) and cumulated percentage volume histograms (PVHs). The ability to differentiate histologic and molecular genetic classes was assessed and compared to volume-of-interest (VOI)-based parameters.

**Results:**

Aggressive WHO grades III and IV and *IDH*-wildtype gliomas were dominated by a high proportion of voxels with an early peak, negative slope, and high TBR, whereby the PVHs with TTP < 20 min p.i., Slope_15–40_ < 0 SUV/h, and TBR_5–15_ and TBR_20–40_ > 2 yielded the most significant differences between glioma grades. We found significant differences of the parameters between WHO grades and *IDH* mutation status, where the effect size was predominantly higher for voxel-based PVHs compared to the corresponding VOI-based parameters. A low overlap of BTV sub-volumes defined by TTP < 20 min p.i. and negative Slope_15–40_ with TBR_5–15 > 2_- and TBR_20–40 > 2_-defined hotspots was observed.

**Conclusions:**

The presented approach applying voxel-wise analysis of dynamic ^18^F-FET PET enables an enhanced characterisation of gliomas and might potentially provide a fast identification of aggressive sub-volumes within the BTV. Parametric 3D ^18^F-FET PET information as investigated in this study has the potential to guide individual therapy instrumentation and may be included in future biopsy studies.

**Electronic supplementary material:**

The online version of this article (10.1186/s13550-018-0444-y) contains supplementary material, which is available to authorized users.

## Background

Structural imaging with T_1_-weighted magnetic resonance imaging (MRI) [1], which is the gold standard in clinical glioma assessment, is restricted to the interpretation of properties like tumour contour, localisation, and enhancement pattern [1]. Besides, several functional MRI techniques have shown relevance for prediction of malignant transformation, involving, e.g. perfusion-weighted imaging (PWI) yielding information on relative cerebral blood volume and flow (rCBV, rCBF) [2–4]. In contrast, positron emission tomography (PET) with amino acids aims to directly image an elevated amino acid metabolism of rapidly proliferating tumour cells [5–7]. According to the report on response assessment in neuro-oncology (RANO), dynamic O-(2-^18^F-fluoroethyl)-l-tyrosine (^18^F-FET) PET has shown its usefulness in diagnosis, in prognosis of tumour progression, and in assessment of treatment response [8].

The current standard procedure for retrieving information from dynamic ^18^F-FET PET consists of evaluating parameters such as the tumour-to-background ratio (TBR) at a certain time point, the late slope, the time-activity curve (TAC) pattern, and the time-to-peak (TTP) [9–16]. In particular, the TTP and the TAC pattern have proven to be suitable for identification of tumour recurrence or progression [12, 13, 17], and for glioma grading [14, 15, 18]. Pharmacokinetic modelling of ^18^F-FET uptake has also been considered. However, to our knowledge, its clinical relevance could not be shown yet, and the requirement of (metabolite-corrected) plasma-input data impairs the clinical applicability [19, 20]. While a slowly increasing TAC is characteristic of low-grade gliomas, the TAC of high-grade gliomas tends to exhibit a short TTP and decreasing TAC [17, 21]. Those parameters are most frequently derived from a mean volume-of-interest (VOI)-TAC of the entire tumour or from the hot-spot of the tumour with a 90% isocontour [17, 22]. However, in case of heterogeneous tumours, it may occur that the hot-spot in summation images does not correspond to the tumour fraction defined as most suspicious regarding tumour aggressiveness according to TTP and TAC pattern. This may potentially lead to an underestimation of malignancy and might impair treatment planning. Recent approaches in current research aiming to improve the assessment of tumour characteristics include, e.g. a slice-by-slice TAC analysis or the extraction of texture parameters from static ^18^F-FET PET images [23, 24].

The goal of this study was to investigate the intra-tumoural distribution of the abovementioned diagnostically relevant kinetic and static parameters derived from dynamic ^18^F-FET PET data on a voxel basis. A comparison with VOI-based methods, as currently utilised for non-invasive glioma characterisation in clinical routine, is provided.

## Methods

### Patients

For this retrospective study we included 162 ^18^F-FET PET positive patients with a newly diagnosed, untreated glioma who had undergone a dynamic 40 min ^18^F-FET PET scan prior to diagnosis according to either biopsy or resection. Both stereotactic biopsy and tumour resection were performed using navigation software (Brainlab iPlan version 3.0, Brainlab, Feldkirchen, Germany). The choice of surgical procedure was based on tumour location, patient age, and performance status as well as patient preference; all treatment decisions have been approved by an interdisciplinary tumour board. Neuropathological diagnosis and grading have been performed by at least two neuropathologists as part of the clinical routine as described previously [18, 25]. Besides histology, mutation of *IDH*1/2 gene and, in case of *IDH*1 mutation, co-deletion of chromosomal material on 1p/19q were analysed in accordance with the recently revised version of the WHO grading system for central nervous tumours [26]. The study was approved by the local ethical review board and all patients gave written informed consent (IRB 606-16).

### Imaging

Dynamic ^18^F-FET PET scans were acquired on an ECAT EXACT HR+ scanner (Siemens Healthineers, Erlangen, Germany) after intravenous bolus injection of 176 ± 13 MBq ^18^F-FET, according to the protocol described in [9, 11]. For patient comfort and minimization of motion during the scan, patients were carefully positioned and fixed. Dynamic 40-min emission data were recorded in 3D mode with 16 frames (7 × 10 s, 3 × 30 s, 1 × 2 min, 3 × 5 min, and 2 × 10 min). Standard corrections for random and scattered coincidences, dead time, decay, and attenuation were performed. Attenuation correction was based on transmission scans measured with three rotating ^68^Ge line sources. Data were reconstructed with filtered back-projection and a 4.9-mm Hann filter. Matrix size was 128 × 128 × 63, and voxel size 2.03 × 2.03 × 2.43 mm^3^. All dynamic PET scans were checked frame-by-frame for head movement. Motion correction was performed on affected time frames within PMOD Fusion tool (v3.5, PMOD Technologies, Zurich, Switzerland).

### Delineation of tumour volume

Biological tumour volume (BTV) was defined by a TBR_20–40_ above 1.6 in static 20–40 min p.i. summation images [15, 27]. Background (BG) values were derived from a crescent-shaped volume of interest (VOI) as described previously [28]. VOIs were defined within the PMOD View tool (version 3.5, PMOD Technologies, Zurich, Switzerland). Only tumour volumes consisting of more than 18 voxels were included, approximating the volumetric PET image resolution.

### Extraction of ‘percentage volume fractions’ and ‘percentage volume histograms’

Voxel-wise analysis was performed with an in-house developed software (C++ with integration of the ROOT data analysis framework, version 6.09/01, Cern, Switzerland; and ITK segmentation and registration toolkit, version 4.11, National Library of Medicine). For each voxel within the BTV, the following kinetic and static parameters were determined: the TTP, the late slope (Slope_15–40_, 15–40 min p.i.), and the tumour-to-background ratios TBR_5–15_ and TBR_20–40_ in early 5–15 min p.i. and late 20–40 min p.i. summation images, with the BG signal derived from the respective time frame. The Slope_15–40_ was estimated by linear fitting of the last three time points, and the TTP was estimated as the time corresponding to the maximal TAC value starting from 2.7 min p.i. to avoid influence from early blood signal. Within the BTV, the sub-volume fractions consisting of voxels with a specific parameter value were determined and stored in histograms. For this, the histograms were plotted with the binned parameter values on the *x*-axis (histogram bin sizes: time frames of dynamic PET images for TTP, 0.6 SUV/h for Slope_15–40_, and 0.25 for TBR) and the percentage fractions of the total BTV on the *y*-axis (percentage volume fractions, PVFs). Cumulated percentage volume histograms (PVHs) were obtained by cumulating these PVF histograms up to the specific bin, to improve the robustness of parameter effect quantification [29, 30]. For example PVF_TTP15–20_ corresponds to the percentage portion of voxels within the BTV with peak value in time frame 14 (15–20 min p.i.), and PVH_TTP < 20_ to the cumulated percentage portion of voxels with TTP < 20 min p.i.. In order to exemplarily illustrate the influence of noise in dynamic PET data onto the estimation of parametric TTP and Slope_15–40_ images, a simple method for noise reduction, a spatial Gaussian filter with 10 mm full width half maximum (FWHM), was applied to the dynamic PET data prior to the estimation and analysis of alternative TTP and Slope_15–40_ images.

### Extraction of VOI-based parameters

For comparison, the following parameters were assessed: TBR_5–15,mean_ and TBR_20–40,mean_ from a mean VOI-TAC (TBR_20–40_ > 1.6) and the maximal TBR_5–15,max_ and TBR_20–40,max_. The VOI for dynamic analysis with TTP and late Slope_15–40_ was obtained with an isocontour set to 90% of maximum uptake in 10–30 min p.i. summation images, yielding a mean TAC characterising the tumour hot-spot [17, 22].

### Statistical analysis

Results are presented as mean value and standard deviation. Statistical analysis was performed with IBM SPSS Statistics (version 24, IBM Corp., Armonk, NY, USA). The threshold for sub-volume fractions defined in the PVH of each derived parameter was optimised by evaluating the overall group differences using the Kruskal-Wallis *H* test. Differences between three groups (molecular genetic sub-groups or WHO grades) were assessed with the Kruskal-Wallis *H* test (effect size, *r* = √(*H*^2^/(*N* − 1), where *H* is the test statistic and *N* the sample size)). This was followed by Dunn-Bonferroni post-hoc analysis for the extraction of significant differences between two groups (effect size, *r* = |*Z*|/√*N*, where *Z* is the *Z* score and *N* the sample size). Receiver-operating characteristics (ROC) analysis was performed in order to determine the cut-off values for distinguishing *IDH*-wt from *IDH*-mut gliomas and WHO grade III/ IV from WHO grade II gliomas. For each test, the threshold (T) yielding the highest product of sensitivity (Se) and specificity (Sp) was chosen as optimal cut-off value. Additionally, *H* test and post hoc analysis were performed for sub-groups separated according to both molecular genetic and histologic features. Differences between WHO grades II and III of *IDH*-mut codel gliomas (i.e. no WHO grade IV) were assessed with Mann-Whitney *U* test.

The similarity between two sub-volume fractions was quantified with the Sørensen-Dice coefficient, i.e. two times the intersection volume divided by the sum of both volumes (2 × (volume_1_ ∩ volume_2_)/(volume_1_ + volume_2_)). Statistical significance was defined as two-tailed *p* value below 0.05.

## Results

### Patients

One hundred twelve patients had a biopsy, and 40 patients underwent a microsurgical tumour resection. In sum, 39 *IDH*1/2-mutant and 1p/19q-codeleted oligodendrogliomas (*IDH*-mut codel), 39 *IDH*1/2-mutant astrocytomas (*IDH*-mut non-codel), 39 *IDH*1/2-wildtype astrocytomas (*IDH*-wt), 6 *IDH*1/2-mutant glioblastomas (GBM *IDH*-mut), and 39 *IDH*1/2-wildtype glioblastomas (GBM *IDH*-wt) were included. Histologic evaluation revealed 55 WHO grade II gliomas, 62 WHO grade III gliomas, and 45 WHO grade IV gliomas. The patient characteristics are given in Table 1.

**Table 1:** Patient characteristics

Patients	162
Gender (f; m)	67; 95
Age (year)	49 ± 15
Procedure for diagnosis	
Biopsy	122
Surgery	40
WHO grade	
II	55
III	62
IV	45
Molecular genetic and histologic classification	
*IDH*-mut, non-codel (WHO II; III)	39 (19; 20)
*IDH*-mut, codel (WHO II; III)	39 (24; 15)
*IDH*-wt (WHO II; III)	39 (12; 27)
GBM *IDH*-mut	6
GBM *IDH*-wt	39

### Statistical analysis

The VOI-based parameters and voxel-based PVHs are presented with respect to WHO grade differentiation (Table 2), molecular genetic differentiation (Table 3), and a combination of both (Table 4). All tables show mean and standard deviation of the parameters. Significance of differences in parameters was predominantly higher for PVH data compared to VOI-based parameters especially in case of molecular genetic differentiation and for differences between WHO grade II and WHO grade III/ IV gliomas. In the following, the respective results for (1) VOI-based and (2) voxel-based analyses are presented. Mean values and results from Kruskal-Wallis *H* test are presented in Tables 2, 3, and 4 with post hoc results coded with upperscript signs (a complete list of results is given in Additional file 1: Table S1, results from ROC analysis are illustrated in Additional file 1: Table S2).

**Table 2:** TTP (units: min p.i.), Slope_15–40_ (units: SUV/h), TBR (units: 1), and BTV_20–40_ (units: mL) from VOI-based analysis and voxel-wise PVH (units: %) separated according to histologic grading

Tumour VOI, post-filtering	Parameter	WHO II (55)	WHO III (62)	WHO IV (45)	*H* test *P*; r	Post hoc
90% isocontour	TTP	25 ± 8	19 ± 9	17 ± 8	< 0.001; 0.39	*°
	Slope_15–40_	− 0.0 ± 0.9	− 0.9 ± 1.6	− 1.0 ± 1.2	< 0.001; 0.36	*°
TBR_20–40_ > 1.6	TBR_5–15,max_	2.9 ± 1.1	3.9 ± 1.6	4.6 ± 1.2	< 0.001; 0.50	*°^#^
	TBR_5–15,mean_	1.8 ± 0.3	2.2 ± 0.5	2.4 ± 0.4	< 0.001; 0.53	*°
	TBR_20–40,max_	2.8 ± 0.9	3.4 ± 1.3	4.0 ± 1.0	< 0.001; 0.43	°^#^
	TBR_20–40,mean_	1.9 ± 0.2	2.1 ± 0.4	2.2 ± 0.3	< 0.001; 0.43	*°^#^
	BTV_20–40_	15 ± 16	26 ± 30	36 ± 25	< 0.001; 0.38	°^#^
	PVH_TBR,5–15 > 2_	25 ± 24	53 ± 27	64 ± 18	< 0.001; 0.55	*°
	PVH_TBR,20–40 > 2_	26 ± 21	37 ± 24	51 ± 17	< 0.001; 0.43	*°^#^
	PVH_TTP > 30_	50 ± 23	32 ± 23	25 ± 15	< 0.001; 0.43	*°
	PVH_TTP < 15_	11 ± 14	26 ± 25	31 ± 15	< 0.001; 0.47	*°
	PVH_TTP < 20_	23 ± 20	45 ± 29	52 ± 18	< 0.001; 0.49	*°
	PVH_Slope < 0_	25 ± 19	46 ± 27	50 ± 17	< 0.001; 0.47	*°
TBR_20–40_ > 1.6, 10 mm Gauss	PVH_GaussTTP > 30_	67 ± 28	41 ± 34	32 ± 23	< 0.001; 0.44	*°
	PVH_Gauss TTP < 20_	13 ± 20	39 ± 34	44 ± 24	< 0.001; 0.51	*°
	PVH_Gauss,Slope < 0_	16 ± 23	45 ± 36	51 ± 26	< 0.001; 0.50	*°

Post hoc *P* < 0.05: WHO grade * II vs. III, ° II vs. IV, ^#^ III vs. IV

**Table 3:** Data shown as in Table 2, separated according to molecular genetic grading

Tumour VOI, post-filtering	Parameter	*IDH*-mut non-codel (45)	*IDH*-mut codel (39)	*IDH*-wt (78)	*H*-test *P*; r	Post hoc
90% isocontour	TTP	25 ± 8	23 ± 9	16 ± 8	< 0.001; 0.45	∆x
	Slope_15–40_	− 0.2 ± 1.5	− 0.2 ± 1.0	− 1.1 ± 1.3	< 0.001; 0.44	∆x
TBR_20–40_ > 1.6	TBR_5–15,max_	3.3 ± 1.5	3.5 ± 1.7	4.2 ± 1.3	< 0.001; 0.37	∆x
	TBR_5–15,mean_	2.0 ± 0.5	2.0 ± 0.5	2.4 ± 0.4	< 0,001; 0.45	∆x
	TBR_20–40,max_	3.2 ± 1.2	3.2 ± 1.4	3.5 ± 1.1	0.060; 0.19	
	TBR_20–40,mean_	2.0 ± 0.3	2.1 ± 0.4	2.1 ± 0.3	0.074; 0.18	
	BTV_20–40_	21 ± 22	28 ± 32	26 ± 24	0.347; 0.11	
	PVH_TBR,5–15 > 2_	32 ± 27	32 ± 26	62 ± 23	< 0.001; 0.52	∆x
	PVH_TBR,20–40 > 2_	33 ± 23	33 ± 25	41 ± 22	0.071; 0.18	
	PVH_TTP > 30_	47 ± 21	50 ± 18	23 ± 20	< 0.001; 0.57	∆x
	PVH_TTP < 15_	12 ± 13	10 ± 9	34 ± 22	< 0.001; 0.56	∆x
	PVH_TTP < 20_	26 ± 20	24 ± 14	56 ± 25	< 0.001; 0.58	∆x
	PVH_Slope < 0_	27 ± 20	25 ± 14	55 ± 23	< 0.001; 0.58	∆x
TBR_20–40_ > 1.6, 10 mm Gauss	PVH_GaussTTP > 30_	62 ± 30	67 ± 24	29 ± 28	< 0.001; 0.55	∆x
	PVH_Gauss TTP < 20_	17 ± 22	12 ± 13	50 ± 31	< 0.001; 0.56	∆x
	PVH_Gauss,Slope < 0_	21 ± 26	15 ± 16	56 ± 32	< 0.001; 0.57	∆x

Post hoc *P* < 0.05: ^+^*IDH*-mut non-codel vs. *IDH*-mut codel, ^∆^*IDH*-mut non-codel vs. *IDH*-wt, ^x^*IDH*-mut codel vs. *IDH*-wt

**Table 4:** Data shown as in Table 2, separated according to molecular genetic and histologic grading

Tumour VOI, post-filtering	Parameter	*IDH*-mut non-codel				*IDH*-mut codel			*IDH*-wt			
		II (19)	III (20)	IV (6)	Post hoc	II (24)	III (15)	*U* test	II (12)	III (27)	IV (39)	Post hoc
90% isocontour	TTP	28 ± 7	24 ± 8 ^∆^	21 ± 11		24 ± 8	22 ± 10		22 ± 10	14 ± 5 ^∆^	17 ± 7	
	Slope_15–40_	0.2 ± 1.0	− 0.4 ± 1.9 ^∆^	− 0.6 ± 1.3		−0.1 ± 0.6	− 0.4 ± 1.5 ^x^		− 0.2 ± 1.3	− 1.5 ± 1.2 ^∆x^	− 1.1 ± 1.2	*°
TBR_20–40_ > 1.6	TBR_5–15,max_	2.9 ± 1.2	3.4 ± 1.7	4.1 ± 1.2	°	2.9 ± 0.9	4.5 ± 2.1	*	3.2 ± 1.2	4.0 ± 1.2	4.7 ± 1.2	°
	TBR_5–15,mean_	1.8 ± 0.4	2.0 ± 0.6 ^∆^	2.2 ± 0.4		1.8 ± 0.2	2.3 ± 0.6	*	2.0 ± 0.4	2.4 ± 0.4 ^∆^	2.4 ± 0.4	°
	TBR_20–40,max_	2.9 ± 1.0	3.2 ± 1.4	3.7 ± 0.9		2.7 ± 0.8	4.0 ± 1.7	*	2.8 ± 1.0	3.1 ± 0.9	4.0 ± 1.0	°^#^
	TBR_20–40,mean_	1.9 ± 0.3	2.1 ± 0.4	2.1 ± 0.2		1.9 ± 0.2	2.3 ± 0.5	*	1.9 ± 0.2	2.0 ± 0.2	2.2 ± 0.3	°^#^
	BTV_20–40_	14 ± 14	22 ± 26	36 ± 21		16 ± 16	47 ± 42	*	17 ± 18	17 ± 19	35 ± 26	°^#^
	PVH_TBR,5–15 > 2_	21 ± 23	37 ± 26 ^∆^	52 ± 27	°	21 ± 20	49 ± 25	*	40 ± 30	67 ± 22 ^∆^	66 ± 16	*°
	PVH_TBR,20–40 > 2_	26 ± 22	36 ± 26	44 ± 14		24 ± 20	48 ± 27	*	27 ± 20	32 ± 21	52 ± 18	°^#^
	PVH_TTP > 30_	57 ± 18	42 ± 20 ^∆^	34 ± 22		51 ± 19	48 ± 17 ^x^		39 ± 32	16 ± 18 ^∆x^	24 ± 13	*^#^
	PVH_TTP < 15_	7 ± 8	14 ± 14 ^∆^	23 ± 14	°	10 ± 9	12 ± 9 ^x^		21 ± 22	44 ± 26 ^∆x^	32 ± 15	*
	PVH_TTP < 20_	17 ± 14	30 ± 20 ^∆^	43 ± 22	°	22 ± 14	27 ± 15 ^x^		36 ± 32	67 ± 25 ^∆x^	54 ± 18	*^#^
	PVH_Slope < 0_	18 ± 15	31 ± 19 ^∆^	43 ± 24	°	23 ± 12	28 ± 15 ^x^		39 ± 29	66 ± 23 ^∆x^	51 ± 16	*^#^
TBR_20–40_ > 1.6, 10 mm Gauss	PVH_GaussTTP > 30_	76 ± 22	55 ± 30 ^∆^	44 ± 35		69 ± 23	64 ± 25 ^x^		49 ± 39	17 ± 27 ^∆x^	31 ± 20	*^#^
	PVH_Gauss TTP < 20_	7 ± 13	22 ± 23 ^∆^	36 ± 26	*°	10 ± 12	17 ± 15 ^x^		28 ± 33	65 ± 32 ^∆x^	46 ± 24	*^#^
	PVH_Gauss,Slope < 0_	9 ± 15	26 ± 28 ^∆^	42 ± 35	°	12 ± 14	21 ± 18 ^x^		34 ± 37	72 ± 32 ^∆x^	52 ± 24	*^#^

Post hoc *P* < 0.05: ^+^*IDH*-mut non-codel vs. *IDH*-mut codel, ^∆^*IDH*-mut non-codel vs. *IDH*-wt, ^x^*IDH*-mut codel vs. *IDH*-wt; WHO grade * II vs. III, ° II vs. IV, ^#^ III vs. IV

### VOI-based parameters

**Fig. 1:**
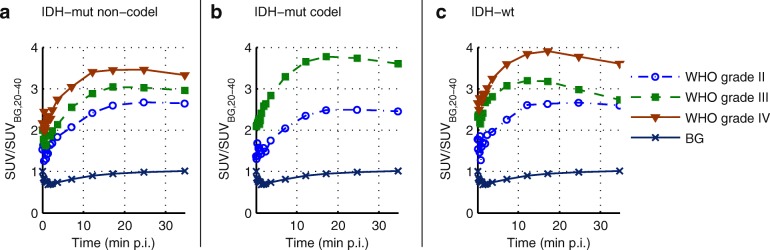
Average over mean time-activity curves of all patients for tumour volumes delineated with a threshold of 90% times maximum activity: **a** *IDH*-mut non-codel, **b** *IDH*-mut codel, and **c** *IDH*-wt

Figure 1 shows the mean TACs of tumour hotspots (90% isocontour) which were used for dynamic analysis separated according to molecular genetic and histologic features. The mean and standard deviation of the parameters are given in the upper parts of Tables 2, 3, and 4.

All considered VOI-based parameters yielded significant differences (*p* < 0.001) between WHO grades (Table 2), with the highest effect size for TBR_5–15,mean_ (*r* = 0.53). TBR_20–40,max_ was not able to differentiate between WHO grade II and III gliomas (*P* = 0.053, *r* = 0.19), and the effect size for TBR_20–40,mean_ was low (*P* = 0.023, *r* = 0.21). The highest effect size for distinguishing WHO grade III from II was found for the TTP (*P* < 0.001, *r* = 0.30, AUC = 0.70, for *T* = 21 min p.i.: Se = 69%, Sp = 67%), and TBR_5–15,mean_ (*P* < 0.001, *r* = 0.37, AUC = 0.76, for *T* = 1.9: Se = 77%, Sp = 67%). The differences between WHO grades II and IV were strongly significant for all parameters (*P* < 0.001) with highest effect size for TBR_5–15,max_ (*r* = 0.49, AUC = 0.86, for *T* = 3.4: Se = 91%, Sp = 78%) and TBR_5–15,mean_ (*r* = 0.51, AUC = 0.87 for *T* = 2.1: Se = 84%, Sp = 80%). TTP, Slope_15–40_, and TBR_5–15,mean_ were not able to differentiate between WHO grades III and IV (*P* = 0.957, *r* = 0.08; *P* = 0.554, *r* = 0.10; *P* = 0.091, *r* = 0.17), and the most significant differences were found for TBR_20–40,max_ (*P* = 0.002, *r* = 0.27, AUC = 0.69, for *T* = 3.0: Se = 80%, Sp = 56%).

Molecular genetic differentiation (Table 3) was strongly significant (*P* < 0.001) for TTP (*r* = 0.45), Slope_15–40_ (*r* = 0.44), TBR_5–15,max_ (*r* = 0.37), and TBR_5–15,mean_ (*r* = 0.45). Differences in TBR_20–40,max_ and in TBR_20–40,mean_ were not significant (*P* = 0.056, *r* = 0.19; *P* = 0.075, *r* = 0.18). None of the parameters differentiated *IDH*-mut non-codel and codel gliomas (*P* > 0.846, *r* < 0.08). Differences between *IDH*-mut non-codel or *IDH*-mut codel and *IDH*-wt gliomas exhibited the highest effect size (with *P* < 0.001) for Slope_15–40_ (*r* = 0.38, AUC = 0.75, for *T* = − 0.4 SUV/h: Se = 74%, Sp = 69%; *r* = 0.34, AUC = 0.75, for *T* = − 0.4 SUV/h: Se = 73%. Sp = 74%) and TBR_5–15,mean_ (*r* = 0.39, AUC = 0.77, for T = 2.1: Se = 78%, Sp = 71%; *r* = 0.35, AUC = 0.76, for *T* = 2.1: Se = 78%, Sp = 79%).

### Percentage volume fractions and percentage volume histograms

**Fig. 2:**
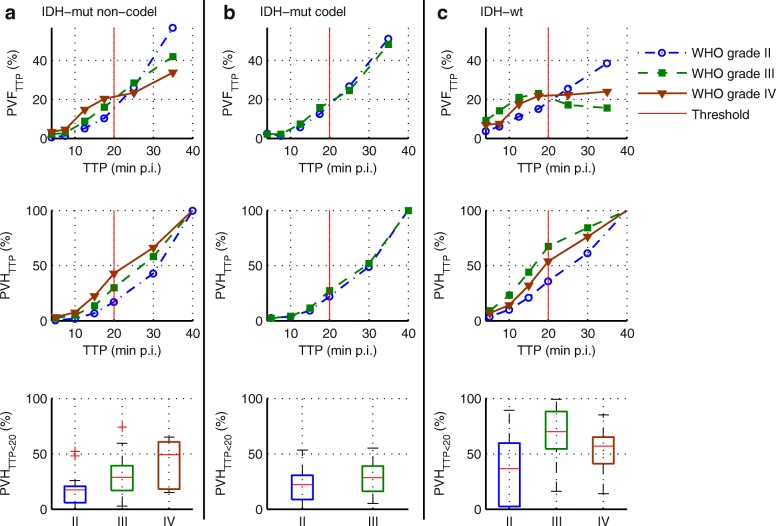
The upper row shows the average percentage volume fractions of the TTP (PVF_TTP_), i.e. the percentage portion of voxels with TTP in the respective time frame. In the middle row, the corresponding cumulated histograms (PVH_TTP_) are presented, i.e. the percentage portion of voxels with TTP below a certain value. The most significant differences between groups were found for PVH_TTP < 20_ (with the cut-off value TTP < 20 min p.i. marked with red lines). The lower row depicts the boxplots of PVH_TTP < 20_. **a** *IDH*-mut non-codel. **b** *IDH*-mut codel. **c** *IDH*-wt

**Fig. 3:**
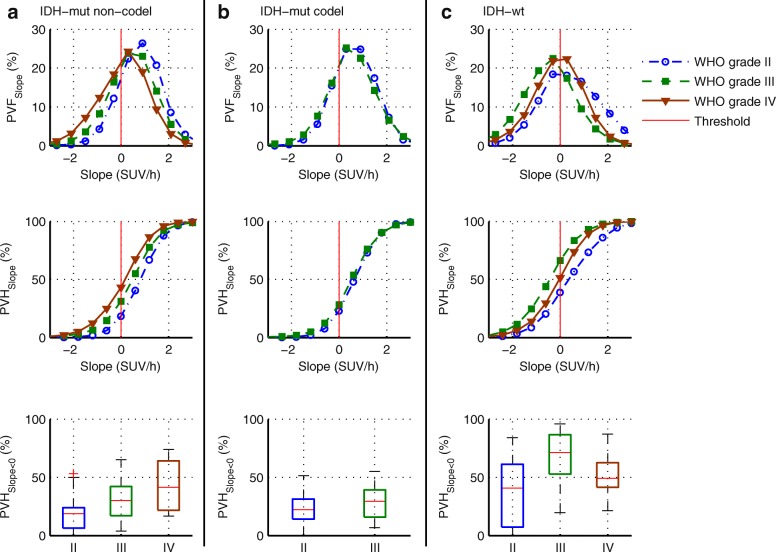
Data presented as in Fig. 2, with average percentage volume fractions of the slope (PVF_Slope_), the corresponding cumulated histograms (PVH_Slope_), and the boxplots of PVH data with slope < 0 SUV/h (PVH_Slope < 0 SUV/h_). **a** *IDH*-mut non-codel. **b** *IDH*-mut codel. **c** *IDH*-wt

**Fig. 4:**
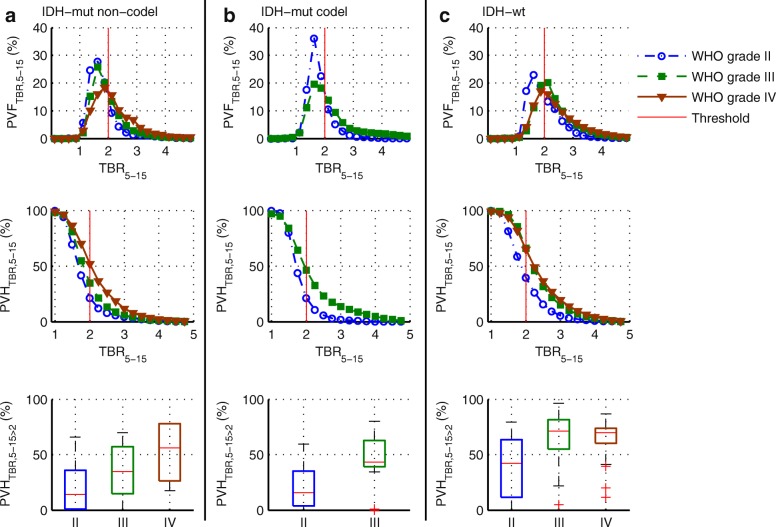
Data presented as in Fig. 2, with average percentage volume fractions of the TBR (PVF_TBR,5–15_), the corresponding cumulated histograms (PVH_TBR,5–15_), and the boxplots of PVH data with TBR_5–15_ > 2 (PVH_TBR,5–15 > 2_). **a** *IDH*-mut non-codel. **b** *IDH*-mut codel. **c** *IDH*-wt

Data from voxel-wise analysis of TTP, Slope_15–40_, and TBR_5–15_ are presented in Figs. 2, 3, and 4. The upper rows depict PVFs, and the middle rows the corresponding cumulated PVFs as PVHs. The red lines represent the PVH cut-offs optimised to yield most significant differences between all glioma entities (minimal *P* value with Kruskal-Wallis *H* test). This resulted in the definition of volume fractions considered to be suspicious of aggressive high-grade characteristics: voxels with TTP below 20 min p.i. (PVH_TTP < 20_), negative Slope_15–40_ (PVH_Slope < 0_), TBR_5–15_ above 2 (PVH_TBR,5–15 > 2_), and TBR_20–40_ above 2 (PVH_TBR,20–40 > 2_) (Tables 2, 3, and 4 and lower rows of Figs. 2, 3, and 4). Additionally, the PVH values for TTP above 30 min p.i. and below 15 min p.i. were included (PVH_TTP > 30_, PVH_TTP < 15_).

All PVH-based parameters showed strongly significant differences between the WHO grades (*P* < 0.001), with the highest effect size for PVH_TBR,5–15 > 2_ (*r* = 0.55) (Table 2). The differentiation of WHO grades II and III and WHO grades II and IV remained strongly significant (*P* < 0.001) for all PVH-based parameters except for PVH_TBR,20–40 > 2_ (WHO grade II vs. III: *P* = 0.022, *r* = 0.21). Effect size was again the highest for PVH_TBR,5–15 > 2_ (distinguish WHO grade III from II: *r* = 0.40, AUC = 0.77, for *T* = 39%: Se = 73%, Sp = 75%; WHO grade IV from II: *r* = 0.53, AUC = 0.89, for *T* = 39%: Se = 91%, Sp = 75%). In contrast, differentiation of WHO grade IV from III was only significant for PVH_TBR,20–40 > 2_ (*P* = 0.007, *r* = 0.24, AUC = 0.66, for *T* = 44%, Se = 69%, Sp = 61%).

All PVH data except PVH_TBR,20–40 > 2_ (*P* = 0.072, *r* = 0.18) yielded strongly significant (*P* < 0.001) differences between molecular genetic groups and remained strongly significant in post hoc analysis of differences between *IDH*-mut (non-codel; codel) and *IDH*-wt gliomas. The highest effect size in post hoc analysis was found for PVH_TTP < 20_ (*r* = 0.47, AUC = 0.82, for *T* = 38%: Se = 77%, Sp = 76%; *r* = 0.47, AUC = 0.86, for *T* = 41%: Se = 74%, Sp = 90%) and PVH_Slope < 0_ (*r* = 0.47, AUC = 0.81, for *T* = 31%: Se = 86%, Sp = 71%; *r* = 0.48, AUC = 0.86, for *T* = 40%: Se = 77%, Sp = 90%).

For a more precise interpretation of the results, glioma types were also separated according to both molecular genetic and histologic features (Table 4). As expected, the mean fraction with early peak (PVH_TTP < 20_) and negative slope (PVH_Slope < 0_) was slightly increased (not significant) in WHO grade IV compared to that in WHO grade III for *IDH*-mut non-codel gliomas. However, in the case of *IDH*-wt gliomas, the fraction of voxels with an early peak (PVH_TTP < 20_: *P* = 0.035, *r* = 0.29) and negative slope (PVH_Slope < 0_: *P* = 0.010, *r* = 0.33) was significantly higher in WHO grade III compared to that in WHO grade IV gliomas. Simultaneously, PVH_TBR,20–40 > 2_ was significantly higher in *IDH*-wt GBMs (*P* = 0.001, *r* = 0.42).

The application of the exemplary Gaussian filter (10 mm FWHM) yielded a comparable ability to differentiate WHO grades and molecular genetic groups, as reported in Tables 2, 3, and 4 and Additional file 1: Tables S1 and S2. However, a tendency of this spatial filtering to reduce the fraction of voxels exhibiting an early peak or negative slope was observed (Additional file 1: Figure S1).

### Spatial correlation of sub-volume fractions

The Sørensen-Dice coefficient, quantifying similarity of the sub-volume fractions, was 0.72 between volumes with TTP < 20 min p.i. and with negative Slope_15–40_, indicating a high overlap of both properties. The Sørensen-Dice coefficients of sub-volumes derived from the static parameter TBR_5–15 > 2_ with sub-volumes derived from kinetic parameters (TTP < 20 min p.i. or negative Slope_15–40_) were 0.50 and 0.48. The corresponding coefficients for the later TBR (TBR_20–40 > 2_) sub-volume were 0.33 and 0.35.

**Fig. 5:**
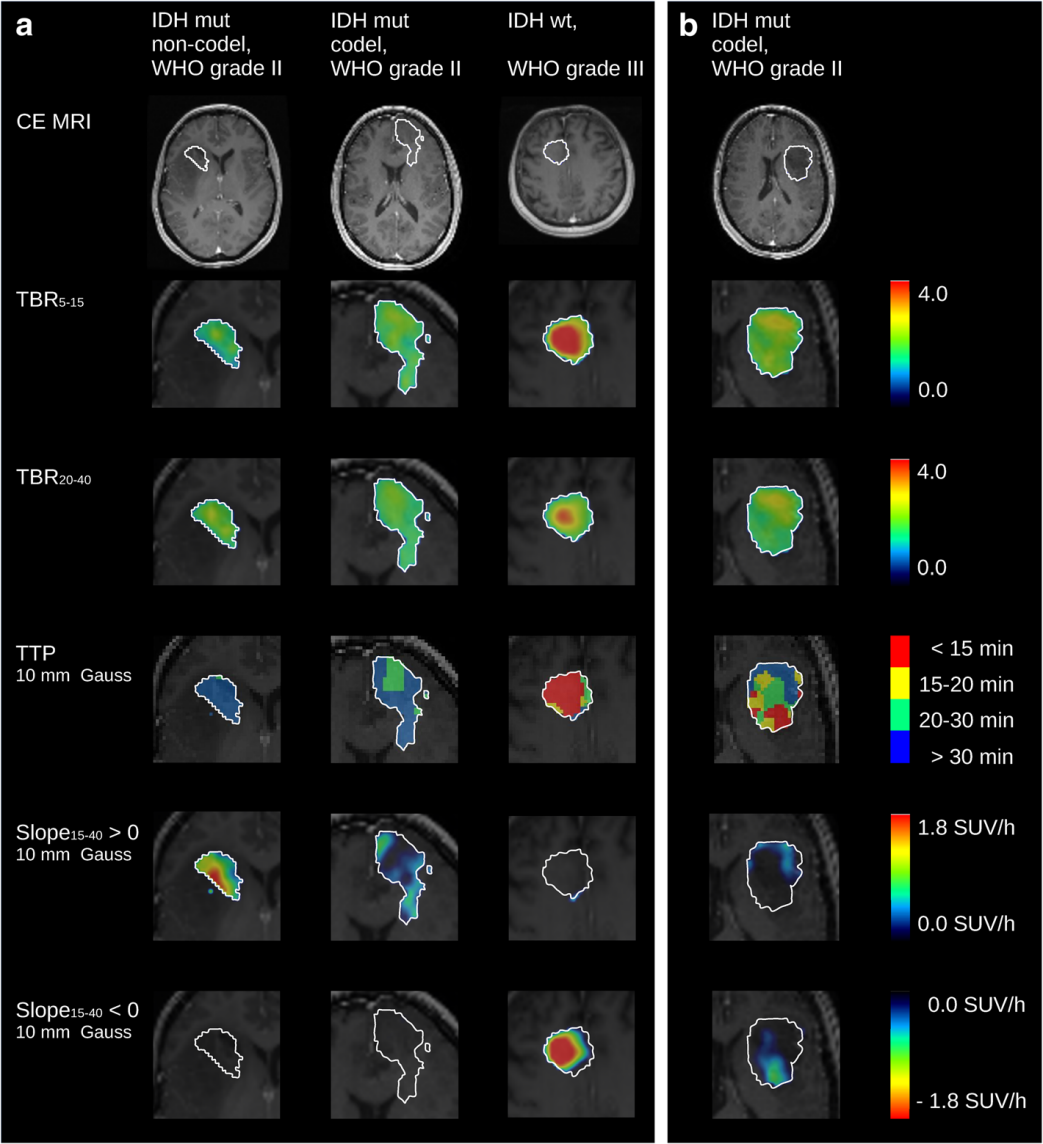
Contrast-enhanced T_1_-weighted MRI images of four example patients, and the corresponding parametric images of the early and late TBR, the TTP, and the negative and positive Slope_15–40_ for the voxels within the BTV (zoom factor 2; BTV marked with white contour; TTP and Slope_5–15_ images estimated from dynamic PET data smoothed with a Gaussian with 10 mm FWHM). **a** Images of three example patients with parameter distributions characteristic of one *IDH*-mut non-codel WHO grade II glioma, one *IDH*-mut codel WHO grade II glioma, and one *IDH*-wt WHO grade III glioma. **b** One example patient (*IDH*-mut codel WHO grade II glioma) with a mixed pattern in parametric images, where maximum uptake in TBR images does not co-localise with the hotspot with early TTP and negative Slope_15–40_

Figure 5 shows the T_1_-weighted MRI images, TBR_5–15_ and TBR_20–40_ images, and parametric maps of TTP and Slope_15–40_ for two typical WHO grade II gliomas (non-codel and codel) and one *IDH*-wt WHO grade III glioma. Additionally, an exemplary tumour with heterogeneous pattern in parametric maps is displayed (classified by biopsy as *IDH*-mut codel WHO grade II glioma), where the maximum uptake in TBR images does not co-localise with the hot-spot in early TTP and negative Slope_15–40_ images.

## Discussion

In this study, we established an automated and reader-independent method for voxel-wise ^18^F-FET PET glioma analysis, which enables a fast identification of sub-volumes consisting of voxels with aggressive high-grade kinetics. By quantifying the intra-tumoural parameter distribution with percentage volume histograms, we found significant differences between WHO grades and between molecular genetic groups. Both, association with WHO grade and *IDH* mutation status, were higher for PVH data compared to VOI-based parameters in most cases. Interestingly, sub-group analyses showed that in the special case of *IDH*-wt gliomas, the fraction with early peak or negative slope was significantly higher in WHO grade III compared to WHO grade IV gliomas, with simultaneously significantly higher PVH_TBR,20–40 > 2_ in WHO grade IV gliomas. Aggressive sub-volumes defined by TTP < 20 min p.i. and negative Slope_15–40_ showed high overlap with each other, but a low overlap with TBR_5–15 > 2_- and TBR_20–40 > 2_-defined hotspots, indicating a possible complementarity of the investigated kinetic and static parameters. The corresponding parametric images as presented in Fig. 5 may provide valuable information for a fast visual screening of glioma tissue. In summary, this study demonstrates the relevance and suitability of tumour heterogeneity assessment on a voxel basis with static and kinetic ^18^F-FET PET parameters for a differentiated characterisation of gliomas, although the clinical applicability of parametric 3D information yet requires a comprehensive validation by utilising stereotactic biopsies.

In this context, an elaborate understanding of the underlying processes of ^18^F-FET uptake is crucial and a matter of current research [20, 31–35]. So far, various studies suggest that regional information from static ^18^F-FET PET images and from MR-based morphological and functional images is complementary, showing only moderate overlap and low spatial correlation [36–39]. Still, tissue properties such as rCBV and rCBF might be relevant for the delivery of ^18^F-FET, potentially influencing ^18^F-FET uptake behaviour. rCBF was found to correlate significantly with early slope (0–5 min p.i.) in ^18^F-FET PET and with TBR (20–40 min p.i.), however, not with TAC patterns and late slope (10–50 min p.i.) [40]. Recently, a negative correlation of rCBV and late slope (10–30 min p.i.) and a positive correlation with TBR (10–20 min p.i.) could be shown; however, only a small fraction of the variance of early and late FET uptake could be explained by rCBV [38]. Therefore, it was concluded that rCBV and ^18^F-FET PET provide congruent and complementary information on the underlying processes. While late TBR may mainly reflect specific trapping within tumour cells, the early TBR and the TAC pattern may be influenced by rCBV and rCBF [38, 41]. Correlation of *IDH* mutation status with MRI parameters has among others shown that *IDH*-wt gliomas tend to exhibit high rCBV values, which is a robust estimate of tumour angiogenesis [32, 35]. In order to retrieve comprehensive information on the underlying processes and their influence on ^18^F-FET uptake, further investigations may combine information from PWI and pharmacokinetic modelling with dynamic ^18^F-FET PET data, also considering blocking studies.

Various studies were published evaluating thresholding techniques optimised for the reproduction of true object boundaries in PET images, possibly taking into account different image characteristics [42–45]. The currently established method for BTV definition was verified with at least one biopsy per patient, which was utilised for an optimisation of sensitivity and specificity and resulted in the optimal TBR cut-off of 1.6 [15, 27]. As shown previously in mice, a threshold relying on background and maximal uptake within the tumour is superior for reproduction of histologically proven glioma boundaries [46]. Hence, future studies considering glioma segmentation in humans, possibly further including information from the characteristic kinetics of the different glioma types, are desirable.

The proposed voxel-wise analysis including TTP and Slope_15–40_ maps and percentage volume histograms of static and kinetic parameters has the potential to provide encompassing information not only for planning of biopsy, surgery, or radiation therapy but also for prognosis, follow-up, and prediction of tumour recurrence based on improved 3D information regarding the local aggressiveness of tumour tissue. In this context, this study has two limitations which need to be addressed in future studies. Firstly, this work would benefit from a correlation analysis of histopathologically assessed tumour heterogeneity and the tumour heterogeneity indicated by the proposed parametric 3D maps. Secondly, voxel-TACs are prone to noise in dynamic PET data, especially for shorter time frames. In this study, sensitive parameters TTP and Slope_15–40_ were derived directly from single-voxel TACs without the application of TAC smoothing or fitting in order to avoid the introduction of bias, i.e. change in temporal pattern, from TAC pre-processing, and allow for an easy adoption by other research centres. An exemplary simple method for per-frame noise suppression with a spatial Gaussian filter was included and showed that PVH data changed while the ability to differentiate glioma types was preserved, which further underlines the need for stereotactic biopsies. Although the incorporation of a kinetic model which is suitable to describe ^18^F-FET pharmacokinetics seems conceivable, provided that appropriate blood input data are available, voxel-based fitting of complex models might also be sensitive to noise [19].

The presented data indicate the direct applicability for non-invasive glioma grading and prediction of molecular genetic profile. This is important, since the WHO classification was updated [26], and stratification is now based on molecular genetic information, i.e. *IDH*-wt gliomas are considered as having the same prognosis as glioblastomas themselves. A direct application is the clinical assessment of lesions suspected of glioma, in particular for the selection of the subsequent clinical steps such as biopsy, resection, or “watch and wait”, but also for risk-stratification in non-contrast-enhancing gliomas (*IDH*-mut vs. *IDH*-wt). The next steps may further include multi-parametric 3D analysis, machine learning approaches, the evaluation of the influence of small scale motion on voxel-wise analysis, and the assessment of the robustness of alternative methods for the voxel-wise characterisation of gliomas, such as pharmacokinetic modelling or the inclusion of information from other imaging modalities like perfusion-weighted imaging.

## Conclusions

Voxel-wise assessment of static and kinetic parameters and partitioning of the entire tumour according to voxel-wise properties enables an improved characterisation of glioma tissue, compared to VOI-based parameters. Moreover, the 3D information might enable a fast visual screening supporting the identification of aggressive sub-volumes, thus guiding individual therapy instrumentation. The correlation between histopathology and the impact on prognosis and prediction of tumour recurrence needs to be evaluated in future studies.

## Additional file

Additional file 1: **Table S1.** *P* values end effect sizes r from post hoc analysis for histologic and molecular genetic differentiation. Effect size *r* is shown colour coded (white-yellow-red continuously scaled from minimal to maximal *r* value). **Table S2.** Area under the curve (AUC) from ROC analysis and the optimal thresholds (*T*) chosen for the highest product of sensitivity (Se, units: %) and specificity (Sp, units: %). Thresholds are given in units of TTP (units: min p.i.), Slope_15–40_ (units: SUV/h), TBR (units: 1), and BTV_20–40_ (units: mL) from VOI-based analysis, and voxel-wise PVH (units: %). AUC is shown colour coded (white-yellow-red continuously scaled from minimal to maximal AUC value). **Figure S1.** Exemplary voxel-wise TACs belonging to the glioma examples shown in Fig. 5. a Voxel-TACs with application of a Gaussian (10 mm FWHM) on dynamic PET data. b Original voxel-TACs without pre-processing of the dynamic PET data. (DOCX 145 kb)

## Abbreviations

BG: Background
BTV: Biological tumour volume
FET: O-(2-^18^F-fluoroethyl)-l-tyrosine
GBM *IDH*-mut: *IDH*1/2-mutant glioblastoma
GBM *IDH*-wt: *IDH*1/2-wildtype glioblastoma
*IDH*-mut codel: *IDH*1/2-mutant and 1p/19q-codeleted oligodendroglioma
*IDH*-mut non-codel: *IDH*1/2-mutant astrocytoma
MRI: Magnetic resonance imaging
PET: Positron emission tomography
PVF: Percentage volume fraction
PVH: Cumulated percentage volume histogram
PWI: Perfusion-weighted imaging
rCBF: Relative cerebral blood flow
rCBV: Relative cerebral blood volume
TAC: Time-activity curve
TBR: Tumour-to-background ratio
TTP: Time-to-peak
VOI: Volume-of-interest
