# Supplementary material for: Voxel-wise analysis of dynamic 18F-FET PET: a novel approach for non-invasive glioma characterisation
Source: EJNMMI Res. 2018 Sep 10;8:91. doi: 10.1186/s13550-018-0444-y (PMC6131687; doi:10.1186/s13550-018-0444-y)
Supplement: Supplementary file 1 — Table S1. P values end effect sizes r from post hoc analysis for histologic and molecular genetic differentiation. Effect size r is shown colour coded (white-yellow-red continuously scaled from minimal to maximal r value). Table S2. Area under the curve (AUC) from ROC analysis and the optimal thresholds (T) chosen for the highest product of sensitivity (Se, units: %) and specificity (Sp, units: %). Thresholds are given in units of TTP (units: min p.i.), Slope15–40 (units: SUV/h), TBR (units: 1), and BTV20–40 (units: mL) from VOI-based analysis, and voxel-wise PVH (units: %). AUC is shown colour coded (white-yellow-red continuously scaled from minimal to maximal AUC value). Figure S1. Exemplary voxel-wise TACs belonging to the glioma examples shown in Fig. 5. a Voxel-TACs with application of a Gaussian (10 mm FWHM) on dynamic PET data. b Original voxel-TACs without pre-processing of the dynamic PET data. (DOCX 145 kb) [file 13550_2018_444_MOESM1_ESM.docx]

**Table S1** *P*-values end effect sizes r from post-hoc analysis for histologic and molecular genetic differentiation. Effect size r is shown colour coded (white-yellow-red continuously scaled from minimal to maximal r value)

| **Tumour VOI, post-filtering** | **Post-hoc** | **Distinguish WHO grade** | | | | | | **Distinguish *IDH*-mut codel** | | **Distinguish IDH-wt from** | | | |
| --- | --- | --- | --- | --- | --- | --- | --- | --- | --- | --- | --- | --- | --- |
|  |  | **III from II** | | **IV from II** | | **IV from III** | | **from *IDH*-mut non-codel** | | ***IDH*-mut non-codel** | | ***IDH*-mut codel** | |
|  |  | ***P*** | **r** | ***P*** | **r** | ***P*** | **r** | ***P*** | **r** | ***P*** | **r** | ***P*** | **r** |
| 90 % iso-contour | TTP | 0.000 | 0.30 | 0.000 | 0.35 | 0.957 | 0.08 | 0.846 | 0.08 | 0.000 | 0.42 | 0.000 | 0.30 |
|  | Slope_15-40_ | 0.002 | 0.27 | 0.000 | 0.35 | 0.554 | 0.10 | 1.000 | 0.02 | 0.000 | 0.38 | 0.000 | 0.34 |
| TBR_20-40_>1.6 | TBR_5-15,max_ | 0.001 | 0.28 | 0.000 | 0.49 | 0.008 | 0.24 | 1.000 | 0.05 | 0.000 | 0.34 | 0.002 | 0.26 |
|  | TBR_5-15,mean_ | 0.000 | 0.37 | 0.000 | 0.51 | 0.091 | 0.17 | 1.000 | 0.02 | 0.000 | 0.39 | 0.000 | 0.35 |
|  | TBR_20-40,max_ | 0.053 | 0.19 | 0.000 | 0.43 | 0.002 | 0.27 | - | - | - | - | - | - |
|  | TBR_20-40,mean_ | 0.023 | 0.21 | 0.000 | 0.43 | 0.007 | 0.24 | - | - | - | - | - | - |
|  | BTV_20-40_ | 0.156 | 0.15 | 0.000 | 0.37 | 0.007 | 0.24 | - | - | - | - | - | - |
|  | PVH_TBR,5-15>2_ | 0.000 | 0.40 | 0.000 | 0.53 | 0.127 | 0.16 | 1.000 | 0.01 | 0.000 | 0.43 | 0.000 | 0.42 |
|  | PVH_TBR,20-40>2_ | 0.022 | 0.21 | 0.000 | 0.43 | 0.007 | 0.24 | - | - | - | - | - | - |
|  | PVH_TTP>30_ | 0.000 | 0.33 | 0.000 | 0.40 | 0.602 | 0.10 | 1.000 | 0.04 | 0.000 | 0.45 | 0.000 | 0.48 |
|  | PVH_TTP<15_ | 0.000 | 0.32 | 0.000 | 0.46 | 0.104 | 0.17 | 1.000 | 0.02 | 0.000 | 0.46 | 0.000 | 0.46 |
|  | PVH_TTP<20_ | 0.000 | 0.36 | 0.000 | 0.46 | 0.249 | 0.14 | 1.000 | 0.02 | 0.000 | 0.47 | 0.000 | 0.47 |
|  | PVH_Slope<0_ | 0.000 | 0.36 | 0.000 | 0.44 | 0.466 | 0.11 | 1.000 | 0.02 | 0.000 | 0.47 | 0.000 | 0.48 |
| TBR_20-40_>1.6, | PVH_GaussTTP>30_ | 0.000 | 0.35 | 0.000 | 0.40 | 0.945 | 0.08 | 1.000 | 0.05 | 0.000 | 0.43 | 0.000 | 0.47 |
| 10 mm Gauss | PVH_Gauss TTP<20_ | 0.000 | 0.39 | 0.000 | 0.48 | 0.392 | 0.12 | 1.000 | 0.03 | 0.000 | 0.45 | 0.000 | 0.47 |
|  | PVH_Gauss,Slope<0_ | 0.000 | 0.39 | 0.000 | 0.46 | 0.518 | 0.11 | 1.000 | 0.04 | 0.000 | 0.45 | 0.000 | 0.48 |

**Table S2** Area under the curve (AUC) from ROC analysis and the optimal thresholds (T) chosen for the highest product of sensitivity (Se, units: %) and specificity (Sp, units: %). Thresholds are given in units of TTP (units: min p.i.), Slope_15-40_ (units: SUV/h), TBR (units: 1), and BTV_20-40_ (units: mL) from VOI-based analysis, and voxel-wise PVH (units: %). AUC is shown colour coded (white-yellow-red continuously scaled from minimal to maximal AUC value)

| **Tumour VOI, post-filtering** | **ROC analysis** | **Distinguish WHO grade** | | | | | | | | | **Distinguish *IDH*-wt from** | | | | | |
| --- | --- | --- | --- | --- | --- | --- | --- | --- | --- | --- | --- | --- | --- | --- | --- | --- |
|  |  | **III from II** | | | **IV from II** | | | **IV from III** | | | ***IDH*-mut non-codel** | | | ***IDH*-mut codel** | | |
|  |  | **AUC** | **T** | **Se, Sp** | **AUC** | **T** | **Se, Sp** | **AUC** | **T** | **Se, Sp** | **AUC** | **T** | **Se, Sp** | **AUC** | **T** | **Se, Sp** |
| 90 % iso-contour | TTP | 0.70 | <21 | 69, 67 | 0.76 | <23 | 78, 67 | 0.56 | <15 | 47, 66 | 0.78 | <23 | 78, 64 | 0.72 | <23 | 78, 51 |
|  | Slope_15-40_ | 0.68 | <-0.4 | 56, 76 | 0.76 | <-0.6 | 67, 84 | 0.57 | <-0.7 | 62, 56 | 0.75 | <-0.4 | 74, 69 | 0.75 | <-0.4 | 73, 74 |
| TBR_20-40_>1.6 | TBR_5-15,max_ | 0.70 | >2.8 | 74, 58 | 0.86 | >3.4 | 91, 78 | 0.68 | >3.5 | 87, 53 | 0.73 | >2.9 | 85, 62 | 0.69 | >3.5 | 68, 69 |
|  | TBR_5-15,mean_ | 0.76 | >1.9 | 77, 67 | 0.87 | >2.1 | 84, 80 | 0.63 | >2.2 | 71, 56 | 0.77 | >2.1 | 78, 71 | 0.76 | >2.1 | 78, 79 |
|  | TBR_20-40,max_ | 0.63 | >2.8 | 56, 67 | 0.82 | >2.8 | 91,67 | 0.69 | >3.0 | 80, 56 | 0.61 | >2.9 | 67, 58 | 0.61 | >3.0 | 60, 67 |
|  | TBR_20-40,mean_ | 0.64 | >1.9 | 56, 69 | 0.82 | >1.9 | 82, 71 | 0.67 | >2.1 | 62, 66 | 0.61 | >1.9 | 72, 51 | 0.60 | >1.9 | 65, 56 |
|  | BTV_20-40_ | 0.61 | >9 | 69, 55 | 0.77 | >9 | 93,55 | 0.68 | >15 | 78, 58 | 0.58 | >9 | 78, 47 | 0.53 | >9 | 78, 56 |
|  | PVH_TBR,5-15>2_ | 0.77 | >39 | 73, 75 | 0.89 | >39 | 91, 75 | 0.62 | >54 | 80, 48 | 0.80 | >49 | 79, 71 | 0.81 | >53 | 76, 79 |
|  | PVH_TBR,20-40>2_ | 0.64 | >31 | 60, 65 | 0.83 | >31 | 87, 65 | 0.66 | >44 | 69, 61 | 0.61 | >29 | 71, 53 | 0.60 | >43 | 54, 67 |
|  | PVH_TTP>30_ | 0.71 | <42 | 68, 67 | 0.81 | <32 | 76, 78 | 0.56 | <28 | 69, 55 | 0.81 | <38 | 82, 71 | 0.85 | <30 | 74, 90 |
|  | PVH_TTP<15_ | 0.71 | >11 | 65, 73 | 0.84 | >10 | 91, 71 | 0.61 | >21 | 76, 55 | 0.81 | >22 | 73, 82 | 0.84 | >21 | 74, 90 |
|  | PVH_TTP<20_ | 0.74 | >28 | 68, 71 | 0.86 | >29 | 91, 73 | 0.59 | >41 | 76, 53 | 0.82 | >38 | 77, 76 | 0.86 | >41 | 74, 90 |
|  | PVH_Slope<0_ | 0.73 | >29 | 69, 69 | 0.84 | >40 | 76, 84 | 0.57 | >42 | 71, 52 | 0.81 | >31 | 86, 71 | 0.86 | >40 | 77, 90 |
| TBR_20-40_>1.6, | PVH_GaussTTP>30_ | 0.72 | <56 | 66, 71 | 0.82 | <47 | 78, 78 | 0.54 | <38 | 71, 50 | 0.79 | <52 | 82, 69 | 0.85 | <48 | 78, 79 |
| 10 mm Gauss | PVH_Gauss TTP<20_ | 0.75 | >10 | 73, 71 | 0.87 | >10 | 91, 71 | 0.57 | >30 | 73, 53 | 0.80 | >31 | 72, 78 | 0.85 | >30 | 72, 90 |
|  | PVH_Gauss,Slope<0_ | 0.75 | >14 | 71, 73 | 0.86 | >18 | 87, 76 | 0.56 | >37 | 73, 52 | 0.80 | >24 | 79, 73 | 0.86 | >40 | 71, 92 |

**Figure S1** Exemplary voxel-wise TACs belonging to the glioma examples shown in Figure 5. **a** Voxel-TACs with application of a Gaussian (10 mm FWHM) on dynamic PET data. **b** Original voxel-TACs without pre-processing of the dynamic PET data
